# Accelerated Infliximab Infusion: Safety, Factors Predicting Adverse Events, Patients’ Satisfaction and Cost Analysis. A Cohort Study in IBD Patients

**DOI:** 10.1371/journal.pone.0166443

**Published:** 2016-11-16

**Authors:** S. Mazzuoli, D. Tricarico, F. Demma, G. Furneri, F. W. Guglielmi

**Affiliations:** 1 Gastroenterology & Artificial Nutrition Dept., “San Nicola Pellegrino” Hospital, Trani (BT), Italy; 2 Department of Pharmacology and Pharmaceutical Sciences, University of Bari “Aldo Moro”, Bari, Italy; 3 Health Economics & Outcome Research Department - EBMA Consulting, Milano, Italy; Case Western Reserve University, UNITED STATES

## Abstract

**Background:**

Standard Infliximab infusion consists of a 2-hour intravenous administration. Recently, Infliximab shortened infusion has been included in the Infliximab label as possible maintenance regimen for patients tolerating Infliximab induction therapy.

**Aim:**

To verify if accelerated 1-hour Infliximab infusions are as safe as standard administrations, in patients with Inflammatory Bowel Disease.

**Methods:**

Seventy-four patients treated between September 2008 and November 2014 were evaluated. Patients were eligible for 1-hour infusion if they had no history of infusion reactions during the previous 2-hour infusions.

**Results:**

Twenty-three patients received 2-hour infusions, 16 patients received 1-hour infusions, 35 patients received 2-hour infusions followed by 1-hour infusions. A total of 1,123 Infliximab infusions were administered. The proportion of patients experiencing infusion reaction was: 4% over the 1-hour infusions and 9% over the 2-hour (P = 0.318). Adverse reaction/infusion rate was 0.55% over the 1-hour infusions and 0.66% over the 2-hour (P = 0.835). In the logistic model, accelerated infusion was the only statistically significant predictor of infusion reaction risk reduction (-90%; P = 0.024). Mean satisfaction was 8/10 (±0.84) with 1-hour regimen and 6/10 (±0.56) with 2-hour infusions (P = 0.000). The mean total cost was reduced by 47% with the 1-hour regimen (133.54€ and 250.86€ for 1-hour and 2-hour infusions, respectively).

**Conclusions:**

Accelerated Infliximab infusion does not increase the acute infusion reaction incidence. In patients with inflammatory bowel disease, the 1-hour regimen should be preferred to 2-hour protocol also due to positive effects on indirect costs and patient’s satisfaction.

## Introduction

Infliximab (IFX) is an effective treatment option for clinical remission and endoscopic healing in Crohn’s Disease (CD) and Ulcerative Colitis (UC) [1–4]. IFX is an intravenous drug recommended to be infused over a two hours (2-h) period followed by one or two hours of clinical observation [5] with a relevant consumption of hospital resources [6,7] and with an infusion reactions (IRs) index observable in 10–20% of Inflammatory Bowel Disease (IBD) patients and in 2.5–5.4% of standard infusion [8]. Current IFX label provides indication that patients who have tolerated (with no IRs) at least 3 initial 2-h IFX infusions (induction phase) and are receiving maintenance therapy, may receive subsequent infusions over a period of 1 hour (1-h) with or without post-infusion monitoring [9–11]. The clinical evidence, showing that the reduction of the infusion duration is safe and does not cause an increase in the number of acute IR, seems to justify the introduction of the IFX accelerated infusion protocol. In the study by Lee et al. in patients with autoimmune diseases [12], the incidence of acute IRs was 0.08 per 1,000 persons/days in the group of 1-h infusion and 0.28 per 1,000 persons/days in the 2-h infusion group (P = 0.070). Recently published cohort studies in a large number of IBD patients have confirmed that 1-h infusions are well tolerated [9,13].

The present analysis aims to assess the safety of shortened IFX infusion, by evaluating the incidence of acute IRs in patients undergoing 1-h and/or 2-h regimen. Moreover, this study aims to evaluate: i) potential predictors of IRs; ii) direct and indirect costs (related with productivity loss) associated with 1-h and 2-h infusions; iii) patient’s satisfaction.

## Materials and Methods

### Patient selection

The present study was approved by the local Ethical Committee of Andria and was conducted using data from a clinical registry developed by the Gastroenterology Department. This was a retrospective, non-randomized, single-center, observational study including 74 IBD patients, treated with IFX between September 2008 and November 2014. All patients were treated in the Gastroenterology Unit of Trani Hospital, according to the current Italian guidelines for the management of Crohn’s Disease (CD) and Ulcerative Colitis (UC) [14]. Patient records were anonymized and de-identified prior to analysis. Demographic and clinical characteristics of the patients, including sex, age, duration of the disease, clinical activity, previous biological treatment, and co-administration of additional immunomodulator drugs (azathioprine -AZA-), methotrexate or steroids, were retrospectively collected. Patients were eligible for 1-h infusion if they had no history of IR during the previous 2-h infusions. Twenty-three patients received 2-h infusions only; 16 patients received 1-h infusions only; 35 patients initially received 2-h infusions, did not report IRs, and then were switched to 1-h infusions. This analysis assessed safety of IFX infusions in the maintenance period only.

### Infusion protocol

In the standard 2-h protocol infusion was initiated at a rate of 75 ml/h for the first 15 minutes, then increased to 200 ml/h for the remaining time (1-h and 45 minutes); patients were then monitored for 2 additional hours after infusion. In the 1-h protocol the infusion was initiated at a rate of 75 ml/h for the first 15 minutes (as in the 2-h protocol), then the rate was increased to 300 ml/h for the subsequent 45 minutes; patients were monitored for 0.5 hours after infusion. All patients received IFX 5 mg/Kg every 8 weeks (q8weeks) and intravenous antihistaminic premedication. Optimization treatment with either an increased IFX dose (10 mg/Kg q8weeks) or a reduction of the infusion interval (5 mg/Kg every 6 weeks-q6weeks-) was adopted in non-responders or in patients losing the response. A specialized nurse, trained on IFX administration, registered infusion data (infusion rate and duration, number of previous infusions) and monitored patient’s vital signs (temperature, blood pressure, oxygen saturation, consciousness and heart rate) during and after infusion. Treatment and observation were interrupted if an acute IR occurred. For IR-free patients, observation ended in November 2014.

### Acute Infusion Reactions

Acute IR was defined as any adverse event occurring during or after an IFX infusion. They were defined as immediate or late reactions (>24 h), according to the onset time, and were classified as: i) mild (flushing, dizziness, headache, palpitation, nausea); ii) moderate (chest tightness, moderate hypo/hypertension >20 points systolic blood pressure, fever, urticaria); iii) severe (symptoms of anaphylaxis like bronchospasm and stridor, dyspnoea, severe hypo/hypertension >40 points systolic blood pressure, fever with one or two hard chills) as reported by Cheifetz [8]. Patients were instructed to report any delayed reactions occurring after an IFX infusion.

### Cost evaluation

For each patient, the average time per type of infusion (1-h and 2-h) was calculated. An average cost of 0.52€/minute for nurse assistance was applied to the entire infusion duration [15]. The same unit cost was used to calculate the cost of the post-infusion monitoring. An average cost of 1.17€/minute, for physician time, was applied to 10 minutes for each infusion (regardless to the regimen) [15]. It was assumed that the specialist was not involved in the monitoring phase. For patients in working age (18–65 years), the cost of productivity loss was also calculated. The average cost of 16.35€/hour was multiplied by 6 hours for 2-h infusions (2 hours for infusion + 2 hours for monitoring + 2 hours for the travelling from/to the hospital) and by 3.5 hours for 1-h infusions (1 hour for infusion + 0.5 hours for monitoring + 2 hours for the travelling from/to the hospital) [16]. Management of severe IR required, on average, 2 hours and 0.5 hours of nurse and physician assistance, respectively. Management of mild and moderate IRs required 15 minutes of assistance from both nurse and physician. All costs were adjusted for inflation to November 2015 [17].

### Patient’s satisfaction

At the end of each IFX infusion, patient’s satisfaction was evaluated. Patients were asked to answer the question: "How much does your current infusion regimen (1-h or 2-h) improve your quality of life?". Answers were codified using a 1–10 Likert scale.

### Statistical analysis

Both descriptive and inferential statistics were calculated to conduct the analysis. Standard descriptive statistics were used to analyse patients’ characteristics at baseline. All continuous variables were expressed as median and interquartile range (IQR: 25^th^-75^th^ percentile) or as mean and standard deviation (SD). Inferential analysis of IRs was performed by conducting both unadjusted and adjusted analyses. Non parametric chi-square test or Fisher’s exact test for binary and discrete variables were used to detect differences between the 1-h and 2-h infusions. For the adjusted analysis, two different approaches were used to determine the predictors of IRs: a patient analysis and an infusion analysis. For the patient analysis, a dataset with patients as observation units was used and a logistic model was run (N = 74). For the infusion analysis, a dataset with administrations as observation units was set up and a generalized estimating equation (GEE) panel model was considered (n = 1,123 infusions for N = 74 patients) [18].

This approach was preferred over generalized linear model (GLM), to take into account: i) dependence among observations (GEE is superior to GLM because key assumptions underlying the use of GLM model include statistical independence of the observations); ii) the increase of “time at risk/exposure” with the number of infusions (panel approach uses the number of infusions as time component). In the economic analysis, costs of all 1-h infusions were compared with costs of all the 2-h infusions. In the patient’s satisfaction analysis, the scores assigned to the two regimens by all the patients who were administered both 1-h and 2-h infusions were compared. A t-test was used to compare satisfaction and costs associated with the two different regimens considered (1-h vs 2-h). Statistical analysis and calculations were performed using STATA software, release 13 (StataCorp. 2015. Stata Statistical Software: Release 13. College Station, TX: StataCorp LP).

## Results

Table 1 shows the patients’ characteristics at baseline.

**Table 1:** Patients’ characteristics at baseline (N = 74 patients).

	Overall (% or IQR)
N	74
Sex	
Male	37 (50%)
Female	37 (50%)
Median Age (years)	45 (33–54)
Disease	
Ulcerative Colitis	29 (39%)
Crohn’s Disease	45 (61%)
Other treatments	
Steroids	68 (92%)
Biologics	14 (19%)
Methotrexate	5 (7%)
Infusions	
10 mg/kg	12 (16%)
Every six weeks	5 (7%)
Concomitant use of AZA	
Yes	39 (53%)
No	35 (47%)
Total number of infusions	1,123
1-h infusions	362
2-h infusions	761
Median number of infusions	15 (7–20)
1-h infusions	7 (0–8)
2-h infusions	10 (5–14)

IQR = interquartile range; AZA = azathioprine.

Two IRs were reported during 1-h infusions and 5 during the 2-h administrations. The proportion of patients experiencing IRs was 3.9% (2 out of 51) during 1-h infusions and 8.6% (5 out of 58) during 2-h administrations (P = 0.318). Considering all infusions, IR rate was 0.55% (2 out of 362) over 1-h infusions and 0.66% (5 out of 761) over 2-h administrations (P = 0.835). Specifically, during 1-h infusions one severe IR (hypotension and bronchospasm) and one mild IR (hypotension) were observed. Both IRs occurred in patients receiving IFX 5 mg/Kg (q8wks). Five IRs were observed in the 2-h group: i) two severe IRs (urticaria, bronchospasm and hypotension); ii) one moderate IR (hypotension); iii) two mild IRs (flushing and palpitation). As regards 1-h administrations, no IRs occurred in patients receiving optimization therapy. As regards 2-h infusions, one patient receiving 10 mg/Kg/q8weeks reported an IR.

Conjoint distributions of potential IR predictors (age, sex, disease duration, concomitant AZA, diagnosis, spondylitis/arthritis, switching to 10 mg/kg regimen, switching to “every 6 weeks” regimen, short infusion regimen, leukapheresis, surgery) and IR incidence were analysed. A statistical difference in the frequency of IRs was found by sex (19% of women reported IR, vs 0% of men, P = 0.000). In the logistic model (Table 2) the short infusion regimen was the only statistically significant predictor of IRs risk reduction (-90%; P = 0.024). None of the remaining independent variables predicted reduction or increase of the IR risk with an acceptable level of statistical significance (P>0.050). The following variables were found to reduce, but only numerically, the IR risk: age< = 40 years; diagnosis< = 7years; simultaneous administration of AZA and IFX; ulcerative colitis; spondylitis/arthritis as concomitant disease; switching to 10 mg/kg regimen; prior surgery treatment.

**Table 2:** Logistic model* to predict the incidence of IRs (N = 74 patients).

Variable	Coefficient	Odds	Δ% Risk	Protective Factor	P-value
Age	-1.02	0.36	-64.05	Age< = 40 vs Age>40	0.295
Disease duration	-0.29	0.75	-25.20	Diagnosis< = 7 years vs Diagnosis>7 years	0.745
Concomitant AZA	-0.31	0.73	-26.59	IFX+AZA vs IFX in monotherapy	0.761
Type of disease	-0.19	0.83	-17.24	UC vs CD	0.848
Spondylitis/arthritis	-0.24	0.79	-20.84	Spondylitis/arthritis Yes vs No	0.855
Switch to 10 mg/kg regimen	-0.97	0.38	-62.15	Switch to 10 mg/kg regimen Yes vs No	0.460
Surgery	-0.73	0.48	-51.77	Surgery Yes vs No	0.619
Short infusion regimen	-2.34	0.10	-90.38	Switch to short infusion regimen Yes vs No	0.024

* Sex and switching to “every 6 weeks” regimen were found to predict exactly the outcome: no men, nor patients undergoing “every 6 weeks” regimen reported IRs. These two predictor were excluded from the analysis.

The GEE panel model confirmed the findings observed in the logistic model (Table 3), except for concomitant use of AZA, switching to 10 mg/kg regimen, and prior treatment with surgery. However, the risk of IRs associated with the infusion duration was only numerically lower in the short regimen group, but statistical significance was not reached (P = 0.218).

**Table 3:** GEE panel model to predict the incidence of IRs (n = 1,123 infusions).

Variable	Coefficient	Odds	Δ% Risk	Interpretation	P-value
Age	-0.88	0.41	-58.50	Age< = 40 vs Age>40	0.252
Short infusion regimen	-1.05	0.35	-64.94	Short vs long	0.218
Disease duration	-0.50	0.60	-39.58	Diagnosis< = 7 years vs Diagnosis>7 years	0.569
Concomitant AZA	-0.61	0.54	-45.83	IFX in monotherapy vs IFX +AZA	0.466
Type of disease	-0.16	0.85	-14.88	UC vs CD	0.847
Spondylitis/arthritis	-0.18	0.83	-16.58	Spondylitis/arthritis Yes vs No	0.877
10 mg/kg regimen	-0.15	0.86	-13.56	5 mg/kg regimen vs 10 mg/kg regimen	0.916
Surgery	-0.11	0.89	-10.83	Surgery No vs Yes	0.907

Regarding the use of resources, the mean time per infusion was 63.6 minutes in the 1-h group and 122.2 minutes in the 2-h group. The mean direct cost of one administration was 78.56€ for 1-h infusions and 156.15€ for 2-h infusions (relative reduction: -49.7%; Table 4). Including costs due to productivity loss in the analysis, mean administration costs were 133.54€ for 1-h infusions and 250.86€ for 2-h infusions (relative reduction: -46.8%). Costs for time spent by nurses and specialists represented the economic driver for both groups (accounting for more than 75% of direct costs and 50% of total costs). Productivity loss costs accounted for about the 40% of total costs. Self-reported patient’s satisfaction was evaluated in 35 patients undergoing 2-h infusions, then switched to 1-h regimen. Mean satisfaction was 8.77/10 (±0.84) with the accelerated modality and 6.44/10 (±0.56) for 2-h infusions (P = 0.000).

**Table 4:** Cost analysis (n = 1,123 infusions).

Type of cost	Cost over all the 1-h infusions (€) (n = 362)	Cost over all the 2-h infusions (n = 761)	Cost per 1-h infusion (€)	Cost per 2-h infusion (€)
Administration	6,523.24	13,713.22	18.02	18.02
Waste disposal	23.33	49.04	0.06	0.06
Specialist during administration	4,235.40	8,903.70	11.70	11.70
Nurse during administration and monitoring	17,630.60	95,792.32	48.70	125.88
Specialist during IRs	17.55	157.95	0.05	0.21
Nurse during IRs	7.80	210.60	0.02	0.28
**Total Direct**	**28,437.92**	**118,826.83**	**78.56**	**156.15**
Loss of productivity due to infusion duration	8,529.62	48,050.02	23.56	63.14
Loss of productivity due to transport from/to the hospital	11,372.82	24,025.01	31.42	31.57
**Total Indirect**	**19,902.44**	**72,075.04**	**54.98**	**94.71**
**Total (Direct +Indirect)**	**48,340.36**	**190,901.87**	**133.54**	**250.86**

## Discussion

In IBD patients tolerating 2-h infusions of IFX scheduled maintenance therapy, the infusion duration can be reduced to 1 hour with good tolerability. This real-world study was designed to evaluate the impact of 1-h IFX infusions in IBD patients. Standard antihistaminic premedication administered to all patients may have positively influenced safety. Overall, IFX infusions were found to be safe: 7 IRs over 1,123 administrations were observed, corresponding to a rate of 0.27% mild reactions, 0.09% moderate reactions, 0.27% severe reactions.

In particular, the accelerated regimen was found to be safe and well tolerated (only 2 IRs over 362 administrations). Shortened infusions did not increase the incidence of IR if compared with 2-h administrations. Unadjusted analysis showed that incidence of IRs was numerically (but not statistically) lower among patients underwent 1-h regimen. The logistic model showed that the risk of IRs was numerically and statistically lower with 1-h infusions (-90%; P = 0.024). The GEE analysis confirmed a risk reduction (-65%) with the shortened infusion (P = 0.218). Despite the statistical significance was not reached with all the three techniques used, our findings suggested that shortened infusions may reduce the risk of IR, if compared with standard regimen. Further studies are required to confirm the trend found with the present analysis. Regarding potential IRs predictors our results show that female patients might have a higher IR risk, compared to men.

The concomitant use of immunomodulators has been recommended to contain the rate of acute IRs [19], but recent data are conflicting [20]. Our multivariate analysis indicated that concomitant use of immunomodulators is not a protective factor.

The cytokine release syndrome (CRS) is one of the possible mechanisms contributing to immediate IR. The CRS is associated with the binding of IFX to the transmembrane TNF alpha in immune cells that activates a cascade of apoptotic signalling which is observed within few hours from the administration with cell degradation and massive release of cytokines in Rheumatoid Arthritis (RA) and CD patients [8, 21]. A shorter infusion duration may interfere with cytokine release for instance reducing the IFX exposure time needed to activate the apoptosis.

A short infusion may also interfere with the complement activation cascade by the anti-IFX antibodies immune complexes. Complement activation cascade has been already reported following IFX infusion [22, 23]. Either CRS and complement activation respond to a reduction in infusion speed as observed in our patients [23, 24].

Anaphylactic reactions resulting from the sudden systemic induction of anti-IFX antibodies of the IgE and IgG isotypes have been rarely reported to contribute to the IFX immediate reactions [8].

Findings of our research suggest that the incidence rate of IRs is limited and comparable with that of all the studies evaluated. Another relevant aspect of this study was the cost saving associated with the accelerated infusion. In the study of Saxena et al. [25], direct costs for nursing assistance decreased of 51.1% per infusion. Our experience confirms that around 60% of the nursing and specialist cost could be saved, with relevant economic benefits for both hospitals and patients. Moreover, reduction of IFX infusion duration would determine positive effects on patient’s satisfaction, offering them more time for productivity and social functioning. This is confirmed by the higher satisfaction scores reported by patients on 1-h regimen, if compared with the 2-h modality.

Overall, the low rate of infusion-related IRs was a clear proof of IFX safety. However, with these limited rates, any statistical proof of the hypothesis of different safety between the two regimens would require a larger sample size than that available for this study. Furthermore, the low IR rate had other implications: i) conduction of IR analysis, stratified by level of severity, was not possible; ii) in the GEE model, very few patients received more than 20 administrations, thus determining an unbalance between number of observations and number of variables, with the latter exceeding the former. In clinical trials, randomization is intended to balance the distribution of both known and unknown confounding factors in the compared groups. This is rarely possible in observational studies and a systematic bias could be generated. Despite the observational design of this study, no adjustment for baseline characteristics (i.e. propensity score) was performed to correct the bias, considering that: i) data came from the same centre; ii) the number of observations was limited. Finally, a selection bias could be also generated as patients who go 1-h infusion are the ones that did not experience previous reactions.

## Conclusions

Despite the above mentioned limitations, the findings of this study suggest that shortened IFX infusion in IBD patients is, at least, as safe as 2-h standard infusion protocols, and determines clear advantages for patient’s satisfaction. In conclusion, our results support the statement that accelerated IFX infusion does not increase the incidence of IRs and is more convenient for patients.

## References

[1] European Medicine Agency. Infliximab: summary of product characteristics; 2015. Available: http://www.ema.europa.eu/docs/en_GB/document_library/EPAR_-_Product_Information/human/000240/WC500050888.pdf. Accessed 26 February 2016.

[2] Mazzuoli S, Guglielmi FW, Antonelli E, Salemme M, Bassotti G, Villanacci V. Definition and evaluation of mucosal healing in clinical practice. Dig Liver Dis 2013; 45:969–77. 10.1016/j.dld.2013.06.010 23932331

[3] Hanauer SB, Feagan BG, Lichtenstein GR, et al Maintenance IFX for Crohn's disease: the ACCENT I randomised trial. Lancet 2002; 359: 1541–1549. 10.1016/S0140-6736(02)08512-4 12047962

[4] Rutgeerts P, Sandborn WJ, Feagan BG, et al IFX for induction and maintenance therapy for ulcerative colitis. N Engl J Med 2005; 353: 2462–2476. 10.1056/NEJMoa050516 16339095

[5] Remicade (Infliximab)-full prescribing information. PA:Centocor Biotech, Inc 2012.

[6] Ollendorf DA, Lidsky L. IFX drug and infusion costs among patients with Crohn’s disease in a commercially-insured setting. Am J Ther 2006; 13: 502–6. 10.1097/01.mjt.0000245223.43783.45 17122530

[7] Wong BJ, Cifaldi MA, Roy S, Skonieczny DC, Stavrakas S. Analysis of drug and administrative costs allowed by U.S. Private and public third-party payers for 3 intravenous biologic agents for rheumatoid arthritis. JMCP 2011; 17: 313–20. 10.18553/jmcp.2011.17.4.313 21534642 PMC10438202

[8] Cheifetz A, Smedley M, Martin S, et al The incidence and management of infusion reactions to infliximab: a large center experience. Am J Gastroenterol 2003; 98: 1315–24. 10.1111/j.1572-0241.2003.07457.x 12818276

[9] Van Assche G, Vermiere S, Noman M et al Infliximab administered with shortened infusion times in a specialized IBD infusion unit: a prospective cohort study. Journal of Crohn’s and Colitis 2010; 4:329–33. 10.1016/j.crohns.2009.12.012 21122522

[10] Breynaert C, Ferrante M, Fidder H, et al Tolerability of shortened infliximab infusion times in patients with inflammatory bowel disease: a single center cohort study. American Journal of Gastroenterology 2011;106:778–85. 10.1038/ajg.2011.61 21407184

[11] Belhassan M, Zeitoun JD, Lefevre JH, et al Infliximab infusion time in patients with inflammatory bowel disease: is longer really safer. Clinics and Research in Hepatology and Gastroenterology 2013; 37:189–92. 10.1016/j.clinre.2012.07.004 23246140

[12] Lee TW, Singh R, Fedorak RN. A one-hour infusion of IFX during maintenance therapy is safe and well tolerated: a prospective cohort study. Aliment Pharmacol Ther. 2011 7;34(2):181–7. 10.1111/j.1365-2036.2011.04699.x 21615434

[13] Babouri A, Buisson A, Bigard MA, Peyrin-Biroulet L. Tolerability of one hour 10 mg/kg infliximab infusions in patients with inflammatorybowel disease. J Crohns Colitis. 2013 3;7(2):129–33. 10.1016/j.crohns.2012.03.007 22472090

[14] National Coordination of chronic patients Associations. Diagnostic and medical assistance patway in chronic inflammatory bowel disease, crohn's disease and ulcerative colitis; 2014. Available: http://www.cittadinanzattiva.it/files/rapporti/salute/malattie_croniche_e_rare/rapporto-pdta-mici-2014.pdf. Accessed 26 February 2016.

[15] Colombo GL, Muzio A, Longhi A. Valutazione economica di Infliximab (Remicade^®^) vs Etanercept (Enbrel^®^) nel trattamento dell’artrite reumatoide. Farmeconomia e percorsi terapeutici 2003; 4 (2).

[16] OECD, Organization economic cooperation development. Average salary; 2014. Available: https://stats.oecd.org/Index.aspx?DataSetCode=AV_AN_WAGE#. Accessed 26 February 2016.

[17] ISTAT, Italian Institute of Statistics. Revaluation Calculator; 2015. Available: http://rivaluta.istat.it/Rivaluta/. Accessed 26 February 2016.

[18] Hanley JA, Negassa A, Edwardes MD, Forrester JE. Statistical analysis of correlated data using generalized estimating equations: an orientation. Am J Epidemiol. 2003 2 15;157(4):364–75. 12578807 10.1093/aje/kwf215

[19] Vermiere S, Noman M, Van Assche G, Baert F, D'Haens G, Rutgeerts P. Effectiveness of concomitant immunosuppressive therapy in suppressing the formation of antibodies to infliximab in Crohn’s disease. Gut 2007; 56:1226–31. 10.1136/gut.2006.099978 17229796 PMC1954977

[20] Neef HC, Riebschleger MP, Adler J. Meta-analysis: rapid IFX infusions are safe. Alimentary Pharmacology and Therapeuticc 2013; 38:365–76. 10.1111/apt.12389 23815183

[21] Urbano PCM, Soccol VT, Azevedo VF. Apoptosis and the FLIP and NF-kappa B proteins as pharmaco dynamic criteria for biosimilar TNF-alpha antagonists. Biologics: Targets and Therapy 2014; 8 211–220. 25114503 10.2147/BTT.S57253 PMC4124053

[22] Karsten CM, Köhl J. The immunoglobulin, IgG Fc receptor and complement triangle in autoimmune diseases. Immunobiology 2012; 217: 1067–1079. 10.1016/j.imbio.2012.07.015 22964232

[23] Lichtenstein L, Ron Y, Kivity S, Ben-Horin S, et al Infliximab-Related Infusion Reactions: Systematic Review. Journal of Crohn's and Colitis 2015; 806–815. 10.1093/ecco-jcc/jjv096 26092578 PMC4558633

[24] Doessegger L and Banholzer ML. Clinical development methodology for infusion-related reactions with monoclonal antibodies. Clinical & Translational Immunology 2015; 4, e39; 10.1038/cti.2015.14 26246897 PMC4524952

[25] Saxena P, Chen G, Jideh B, Collins G, Leong RW. Safety and cost benefit of an accelerated infliximab infusion protocol in the treatment of ambulatori patients with inflammatory bowel disease. Expert Opinion on Biological Therapy 2014; 14:277–82. 10.1517/14712598.2014.866649 24359522

